# The Role of 3D Printing in Customizing Dental Prosthetics and Orthodontic Appliances

**DOI:** 10.31661/gmj.v13iSP1.3719

**Published:** 2024-12-30

**Authors:** Fatemeh Abedi Diznab, Hooman Ghazi Oskouei, Faeze Dehghan, Mohamad Dehghan, Mohammad Golrokhian, Ali Rafighi, Naghmeh Shenasa

**Affiliations:** ^1^ Department of Orthodontics, School of Dentistry, Tehran University of Medical Sciences, Tehran, Iran; ^2^ Department of Orthodontics, School of Dentistry, Ahvaz Jundishapur University of Medical Sciences, Ahvaz, Iran; ^3^ Faculty of Dentistry, Islamic Azad University of Medical Sciences, Tehran, Iran; ^4^ Specialist in Prosthodontics, Independent Researcher, Tehran, Iran; ^5^ Department of Oral and Maxillofacial Surgery, School of Dentistry, Isfahan University of Medical Sciences, Isfahan, Iran; ^6^ Department of Orthodontics, Faculty of Dentistry, Tabriz University of Medical Sciences, Tabriz, Iran; ^7^ Private Practice, Shahrekord University of Medical Science,Endodontics Department, Shahrekord, Iran

**Keywords:** 3D Printing, Prosthetics, Orthodontic

## Abstract

3D printing technology has introduced significant advancements in dentistry, particularly in the customization of dental prosthetics and orthodontic appliances. By enabling precise, patient-specific designs, 3D printing enhances both the fit and comfort of dental devices, improving patient outcomes and satisfaction. This technology offers notable efficiencies over traditional manufacturing methods, reducing production times and costs while supporting seamless digital workflows in clinical practice. Recent advancements in biocompatible materials and digital integration have expanded the application of 3D printing to a range of dental devices, from crowns and bridges to clear aligners and retainers. However, challenges remain, including material limitations, regulatory hurdles, and technical constraints that can impact adoption, particularly in smaller clinics. Future research aims to address these challenges by exploring new materials, incorporating artificial intelligence for optimized design, and enhancing environmental sustainability through waste reduction. The ongoing evolution of 3D printing in dentistry promises to further personalize and streamline dental care, paving the way for a more patient-centered, efficient, and accessible approach in modern dental practices.

## Introduction

The advent of 3D printing has marked a transformative period across many fields, notably in healthcare, where its role in dentistry has shown tremendous promise [1]. As digital technologies continue to reshape patient care, 3D printing, also known as additive manufacturing, allows for the production of highly customized, patient-specific devices [2]. This approach is particularly beneficial in dental prosthetics and orthodontic applications, where precision, fit, and customization are paramount [3]. By creating intricate structures layer by layer, 3D printing technology can produce complex dental components with unmatched accuracy, helping to streamline processes, enhance treatment outcomes, and reduce the time and costs associated with traditional methods [4].

The origin of 3D printing technology dates back to the 1980s, with its initial applications in the prototyping and manufacturing sectors [5]. However, its incorporation into healthcare began to gain traction in the early 2000s, with dentistry emerging as a particularly fertile ground for innovation [6]. Traditional methods for producing dental prosthetics and orthodontic appliances involve several steps, from manual impressions and model casting to complex laboratory processes, which often result in variability and may be time-consuming [7]. In contrast, 3D printing allows for direct digital-to-physical transitions, enabling clinicians and dental technicians to manufacture crowns, bridges, dentures, and orthodontic appliances directly from digital impressions [8].

This review aims to provide a comprehensive understanding of its application in dental prosthetics and orthodontics, offering insights into recent research, future directions, and emerging challenges.

## 3D Printing Technologies Used in Dentistry

**Figure-1:**
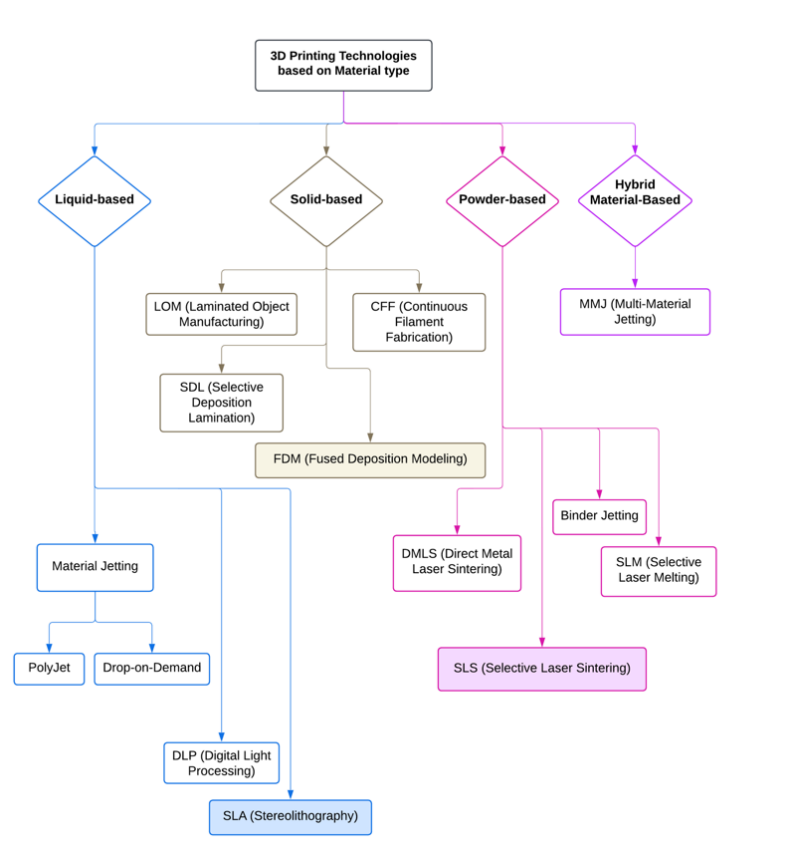
Figure 1. Classification of 3D printing technologies

**Table:** Table1. Comparison of 3D Printing Technologies for Customizing Dental Prosthetics and Orthodontic Appliances

**Technology**	**Liquid-based** ( **SLA)**	**Solid-based (FDM)**	**Powder-based** **(SLS)**
**Print Resolution (µm)**	30-150	200-400	200
**Layer Thickness (µm)**	25-100	178 or 254	30-100
**Material**	Photopolymer resin	Thermoplastic filament (PLA, ABS, TPU, ASA)	Powder (Co-Cr, titanium, PEEK, polyamides)
**Flexural Modulus** **(MPa)**	1456 - 1654	1750 - 3185	345 -551
**Flexural Strength** **(MPa)**	19-135	83 - 102	19-86
**Tensile** **Modulus (MPa)**	18-901	1000 - 1538	40-1700
**Tensile** **Strength (MPa)**	1- 60	31 - 71	1-62
**Elongation (%)**	0.06 - 11	4-5	2-122
**Cost**	Low to medium	Low	Very high
**Advantages**	-High resolution -Smooth finish -Precise fit	-Affordable access -Broad materials -Ideal for prototyping	-High strength -Complex geometries -No supports needed
**Limitations**	- Needs post-processing - Limited biocompatibility	-Low resolution -Limited aesthetics	-Expensive equipment -High cost
**Applications**	- Crowns, bridges, and inlays - Orthodontic models and molds - Clear aligners and retainers - Orthodontic devices	-Educational use - Surgical guides - Preliminary models - Non-critical dental parts	-Denture frameworks -Metal substructures

**μm:** micrometer equal to 0.001 mm, **MPa:** Megapascal

In the field of dentistry, a variety of 3D printing technologies have become central to the production of customized prosthetics and orthodontic appliances, each offering distinct advantages based on their technical specifications and material compatibility [9].

Among the most widely used methods are stereolithography (SLA), fused deposition modeling (FDM), and selective laser sintering (SLS) [10][11]. On the other hand, Material innovation has played a key role in advancing 3D-printed dental prosthetics.

3D Printing Techniques

3D Printing Techniques vary in their approach to additive manufacturing, with differences in precision, and suitability for specific dental applications [12]. Understanding the principles, strengths, and limitations of each technology can help determine their optimal uses in clinical settings [13].

These technologies are commonly classified by material type including solid, liquid, powder, and hybrid. Solid-based materials such as thermoplastics in fused deposition modeling (FDM) create robust models and retainers (Figure-1) [9][14]. Liquid-based photopolymers used in stereolithography (SLA) and digital light processing (DLP) provide high precision for aligners and detailed prosthetics. Powder-based ceramics and metals in selective laser sintering (SLS) and melting (SLM) are ideal for strong, custom frameworks in crowns and implants. Each type offers unique strengths for tailored dental solutions [9][10][11]. Hybrid Material-Based such as Multi-Material Jetting (MMJ) is a new technology that allows for the combination of materials, often with varying properties within a single print, and it is highly suitable for educational model production.[14][15] Table-1 demonstrates a comparison of SLA, FDM, and SLS as the examples of common 3D printing technologies that are used in customizing dental prosthetics and orthodontic devices [2][9][10].

SLA Technology

SLA is one of the most established 3D printing technologies, known for its high-resolution capabilities and ability to produce smooth surface finishes [16]. In SLA, a laser selectively cures layers of liquid photopolymer resin, creating highly detailed structures with fine accuracy [16][17]. This level of precision makes SLA ideal for producing custom dental prosthetics, such as crowns, bridges, and inlays, which require detailed anatomical accuracy [18].

SLA is also commonly used for creating orthodontic models and molds due to its capacity for fine details, the average error in full-arch dental models is between 3.3 μm and 579 μm [19]. Also, SLA is known for very high precision, capable of achieving resolutions as fine as 30-150 µm [10]. However, the materials used in SLA, primarily photopolymer resins, may require post-processing and curing to achieve optimal strength, and some may not fully meet biocompatibility standards for long-term intraoral use [20].

DLP is similar to SLA in that it also uses a photopolymer resin but employs a digital light projector to cure an entire layer of resin simultaneously rather than a laser tracing each layer [21][22]. This approach enables faster printing speeds compared to SLA, making DLP advantageous for high-demand clinical environments where quick turnaround times are critical [23][24]. DLP’s high resolution and efficiency make it well-suited for producing clear aligners, retainers, and other orthodontic devices that require precise fit and durability [21][23]. While the materials for DLP are similar to those used in SLA, advancements in DLP-compatible biocompatible resins continue to expand their applicability in directly printed dental devices [25].

FDM Technology

FDM, also known as fused filament fabrication (FFF), operates by extruding thermoplastic filaments layer by layer to build up the desired structure [26]. In dentistry, FDM is often used to create preliminary models, surgical guides, and other non-critical dental parts where high resolution is not essential [27]. Some thermoplastic materials used in FDM, such as PLA (polylactic acid) and ABS (acrylonitrile butadiene styrene), can be biocompatible, though their mechanical properties and aesthetics may limit their use in long-term intraoral applications [12][28]. FDM’s cost-effectiveness makes it accessible to smaller practices for prototyping and training purposes, despite its lower resolution [29].

SLS Technology

SLS uses a laser to sinter powdered materials, such as nylon or metal, layer by layer, to form a solid structure [30]. This technology allows for the production of robust, durable components, making SLS an excellent choice for applications that require mechanical strength, such as partial denture frameworks or metal substructures for crowns and bridges [31]. Unlike SLA, SLS does not require support structures during printing, allowing for more complex geometries and reduced material wastage [30]. While SLS offers significant durability, it typically requires more expensive equipment, which may limit its use to larger dental laboratories or specialized practices [32][33]. Also, SLS is limited to processing certain polymers and metals, with limited capability to process ceramics and composites effectively. This restricts material choices for dental applications, where ceramics are often desired for aesthetic and biocompatible properties [34].

Material Considerations

Table-2 provides a comparison of materials used in 3D-printed prosthetics, highlighting key attributes such as biocompatibility, strength, and specific application areas. Biocompatible resins and metal powders have emerged as primary materials in 3D printing, meeting the high standards for durability, aesthetics, and patient safety in oral applications [35]. Photopolymer resins, used in SLA, offer high-resolution capabilities and are ideal for producing crowns and bridges with intricate details and a natural appearance [36]. In contrast, metal powders, used in SLS and selective laser melting (SLM), provide strength and durability, making them suitable for dental implants and partial denture frameworks that need to withstand significant functional stresses [36][37]. Advances in hybrid materials, such as reinforced polymers and composite resins, are also contributing to the enhanced durability and esthetic appeal of 3D-printed prosthetics, addressing patient demand for both functionality and appearance [36][38].

## Applications in Dental Prosthetics

**Figure-2:**
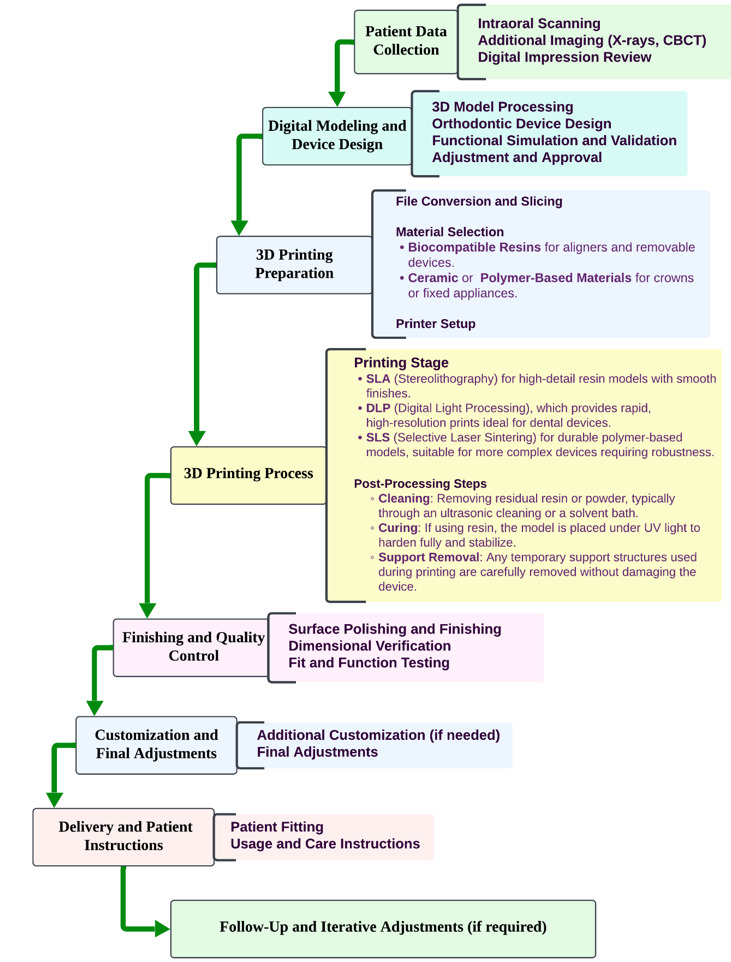
Figure 2. Workflow Of 3D-printed orthodontic devices and dental prosthetics

**Table:** Table2. Materials for 3D-printed Dental Prosthetics and Orthodontic Appliances

**Category**	**Material Type**	**Biocompatibility**	**Strength**	**Durability**	**Typical Use**
	BioMed Amber Resin	High, suitable for surgical use	High	High	Surgical guides, implant placements
**Prosthetics**	Cobalt-Chrome Alloy	High, well-suited for permanent use	Very high	Very high	Customized lingual orthodontic appliances, prosthetics
	PolyJet Resins (e.g., VeroWhite/VeroClear)	Moderate to high	Moderate	Moderate to high	Dentures, temporary crowns, and trial prostheses
	Dental LT Clear Resin	High, suitable for intraoral use	Moderate	Moderate	Aligners, retainers, and surgical guides
**Orthodontics**	Tera Harz TC-85	Specifically marketed for aligners	Moderate	High	Clear aligners for orthodontic treatments
	Polyurethane	High, tested in cytotoxicity and hemolysis	Moderate	Moderate	Clear aligners, splints
	PET-G (Polyethylene Terephthalate Glycol)	Moderate biocompatibility	Moderate	Moderate	Clear aligners and orthodontic models

The application of 3D printing in dental prosthetics has revolutionized the production of custom crowns, bridges, dentures, and implants, allowing for levels of personalization and precision previously unattainable with traditional manufacturing methods [33]. Figure-2 illustrates a comprehensive workflow for producing custom 3D-printed orthodontic devices and dental prosthetics.

This technology enables the rapid and accurate creation of patient-specific prosthetics, enhancing both functional outcomes and patient satisfaction [39]. By using digital impressions and computer-aided design (CAD) software , dental professionals can now design and produce prosthetics that closely match the unique anatomical features of each patient, leading to superior fit and comfort [1].

One of the primary benefits of 3D printing in dental prosthetics is its ability to deliver highly customized devices [40]. The process begins with digital scanning of the patient’s oral structures, allowing for exact digital replicas of crowns, bridges, and dentures to be designed based on the patient’s specific dental morphology [41]. This approach reduces the margin for error inherent in manual impression-taking and model-casting processes [42]. Consequently, prosthetics created through 3D printing often exhibit greater precision and adaptability, which not only improves function but also reduces the number of adjustments required during fitting, ultimately streamlining the overall treatment process [33].

Clinical studies further validate the impact of 3D printing on dental prosthetics. For instance, a recent study on 3D-printed crowns demonstrated that patient-specific crowns created with 3D printing achieved better fit and retention compared to conventional methods, reducing the likelihood of future adjustments and repairs [43].

Chen et al, [44] demonstrated that 3D-printed designs could reduce peak contact pressure on oral tissues by 70%, significantly improving patient comfort and reducing the risk of mucosal lesions and pressure-induced sores and improving uniformity by 63%, potentially reducing long-term residual ridge resorption.

Another study examined the effectiveness of 3D-printed denture bases and found that they provided improved adaptation to the soft tissues in the mouth, resulting in enhanced patient comfort reduced incidence of pain, and improved esthetics and function [45].

These applications illustrate the transformative potential of 3D printing in dental prosthetics. By offering a customizable, efficient, and patient-centered approach, 3D printing is setting new standards in prosthetic dentistry, delivering improved outcomes that benefit both clinicians and patients [42].

## Applications in Orthodontic Appliances

The use of 3D printing in orthodontics has expanded significantly, particularly in the production of clear aligners, retainers, and other custom devices that facilitate precise and patient-specific treatments [46]. Figure-1 shows the workflow for producing custom 3D-printed clear aligners, which have become a popular alternative to traditional braces, and are one of the most notable advancements in 3D-printed orthodontics [47]. By leveraging digital designs, 3D printing enables the production of aligners that are tailored to the specific dental structure of each patient, ensuring optimal fit and comfort [48]. The customization process allows orthodontists to create a series of aligners that progressively guide teeth into alignment according to a carefully mapped treatment plan [49]. Also, 3D printing is used to manufacture retainers and other custom orthodontic appliances, offering patients comfortable, well-fitted devices that enhance both the effectiveness and convenience of orthodontic treatment [50].

The integration of digital scanning and 3D printing has also contributed to a more seamless workflow in orthodontic treatment [51]. Using intraoral scanners, clinicians can capture detailed, 3D images of a patient’s teeth and gums [52]. These scans are then used to create digital models, which serve as the basis for designing aligners or retainers [53]. Once the design is finalized, it is sent directly to a 3D printer for fabrication. This streamlined workflow reduces reliance on physical impressions, which can be uncomfortable for patients and prone to error [23][54]. By enhancing the accuracy and efficiency of treatment planning, digital workflows improve the overall quality of orthodontic care and reduce the time patients spend waiting for appliances [55].

Through advancements in customization, precision, and digital integration, 3D printing is transforming orthodontic care [49]. This shift towards a digital, patient-centered approach underscores the value of 3D printing in modern orthodontics, positioning it as a core technology in the future of dental care [48].

## Clinical Impact and Benefits

The integration of 3D printing into dental practices has demonstrated substantial clinical benefits in the field of prosthetics and orthodontics [8]. By providing tools for highly personalized and precise dental solutions, 3D printing has helped redefine both the patient experience and the operational landscape for dental clinics [56].

Accuracy

The accuracy of 3D-printed prosthetics and orthodontic appliances is a critical factor in their clinical effectiveness. This method can achieve a high level of precision, contributing to more predictable treatment outcomes [9]. Also, Several reports show that high-resolution 3D printers can produce dental models with dimensional deviations within clinically acceptable limits, allowing for precise fitting of prosthetic components and orthodontic devices [9][23][57]. For instance, 3D-printed models for fixed dental prostheses, despite slight deviations from conventional stone casts, still meet the accuracy required for clinical use [58]. Similarly, studies on orthodontic retainers printed from digital impressions confirm high reproducibility and reliability in fit, which is comparable to traditional thermoformed retainers[9][10]. Although some variations in trueness and precision exist depending on the printer type and settings, 3D printing generally provides the accuracy needed for both prosthetic and orthodontic applications in dentistry [59].

Patient Satisfaction

Research highlights the benefits of 3D printing in improving patient comfort, customization, and satisfaction for dental prosthetics and orthodontic appliances [60]. Some studies demonstrate that 3D-printed dental appliances allow for a high degree of personalization, leading to improved fit and function while reducing the number of adjustments needed [33][47]. This precision not only shortens treatment time but also enhances overall patient satisfaction and reduces the discomfort associated with traditional manufacturing processes [45][61]. Moreover, 3D printing’s efficient workflow can reduce the number of office visits and speed up the time needed for fabrication, which is reflected in patient reports of faster treatment initiation and high satisfaction levels [35].

In contrast, some researchers reported, that patient satisfaction with traditional dentures was higher in areas such as speech, ease of cleaning, stability, comfort, and overall contentment. Meanwhile, digital dentures produced via 3D printing show similar practicality and effectiveness to conventional options, with 20% of patients favoring them for daily use [62].

Cost Savings

A key benefit of 3D printing in orthodontics lies in its precision and cost-effectiveness [63]. Unlike traditional methods, which involve labor-intensive steps such as casting molds and adjusting wire-based components, 3D printing allows for the direct production of aligners and retainers from digital models [64]. A study found that using 3D printing to produce digital dentures at a university dental clinic resulted in 18% cost reductions, as fewer appointments and material use were required compared to conventional laboratory-fabricated dentures [65].

Time Reduction

This digital-to-physical process not only improves the accuracy of fit but also significantly reduces production time [66]. The faster production timelines made possible by 3D printing not only improve the clinic’s operational efficiency but also allow for a more responsive approach to patient care, enhancing the clinic's capacity to manage higher patient volumes with minimal delays [67]. Ballard et al.,[68] demonstrated using 3D-printed surgical guides for dental implant placement and maxillofacial surgeries reduces procedural time significantly. This includes a reduction of approximately 23 minutes per procedure, translating to substantial time and cost efficiency.

In the realm of educational implications, 3D printing is transforming dental education and training, offering students and practitioners hands-on experience with digital design and additive manufacturing [6]. Traditional dental education often relies on physical models and manual techniques, which can be limited in scope and scalability [69]. With 3D printing, students can gain practical experience in creating digital models and printing custom prosthetics and orthodontic appliances, equipping them with skills that align with current technological advancements in dental practice [42].

Moreover, 3D printing allows for the production of realistic anatomical models for practice, enabling students to develop procedural skills in a controlled, hands-on environment without the need for live patients [35]. This capability not only improves educational outcomes but also enhances the ability of dental schools to simulate complex cases, helping to prepare future practitioners for real-world clinical challenges [4].

Environmental Benefit

One of the primary environmental benefits of 3D printing in dentistry is material efficiency [70]. Traditional manufacturing of dental prosthetics often involves subtractive methods, where materials are cut or shaped from larger blocks, resulting in a significant amount of wasted material [71]. Milling a dental crown from a ceramic block or creating aligners from multiple molds can lead to excess waste, much of which is non-recyclable [72]. In contrast, 3D printing builds objects layer by layer, using only the exact amount of material needed for each device [73]. This process not only conserves materials but also minimizes waste, reducing the environmental impact of manufacturing dental devices [74].

3D printing also offers energy efficiency advantages, particularly when compared to conventional manufacturing, which often requires multiple machines, molds, and energy-intensive processes [75]. 3D printing, however, consolidates these steps into a single process, significantly lowering the overall energy consumption [47]. By using digital workflows, dental professionals can produce devices with fewer machine operations, reducing the carbon footprint associated with energy use in production facilities and laboratories [76].

Another aspect of 3D printing's environmental sustainability in dentistry is the potential for recyclable and biodegradable materials [77]. Advances in 3D printing materials have led to the development of recyclable resins and biodegradable polymers, which could further reduce the environmental impact of dental manufacturing [78]. Ongoing research into biodegradable polymers may allow dental devices to break down more naturally at the end of their lifecycle, offering an eco-friendly alternative to traditional materials that remain in landfills [79].

While 3D printing presents promising environmental advantages, it is not without challenges. Post-processing requirements for 3D-printed devices can involve cleaning, curing, and finishing steps that may use additional chemicals and generate waste [70]. Supports and excess resin used in SLA printing need to be removed and properly disposed of, which can diminish the overall sustainability of the process [78]. Also, certain materials, especially high-strength resins and metal powders, may not yet be fully recyclable, limiting the environmental benefits of 3D printing in some applications [73]. Overall, by adopting 3D printing, dental practices can contribute to a more sustainable healthcare sector, aligning with the broader global movement toward eco-friendly practices in medicine and beyond.

## Challenges and Limitations

Despite its numerous advantages, the application of 3D printing in dentistry also faces several challenges and limitations. These issues, which range from material and regulatory constraints to technical limitations and adoption barriers, highlight the complexities of integrating this technology into clinical practice [80].

Material constraints remain one of the most significant challenges in 3D-printed dental devices. Dental applications require materials that are not only durable but also biocompatible and aesthetically suitable, particularly for devices [81]. While advances in 3D printing materials, such as photopolymer resins and metal powders, have expanded options for dental use, limitations still exist in terms of strength, color stability, and long-term durability [73]. For example, some biocompatible resins used in SLA printing may lack the necessary resilience for high-stress applications or may degrade over time in the oral environment [82]. Additionally, achieving a natural appearance that matches the patient’s existing dentition remains a challenge for some 3D printing materials, which may affect patient satisfaction and limit the applicability of 3D printing for highly visible dental prosthetics [83].

Regulatory challenges are another critical barrier to the widespread adoption of 3D-printed dental devices [84]. Given the direct contact these devices have with oral tissues, stringent standards are essential to ensure safety, biocompatibility, and effectiveness [85]. Regulatory agencies such as the U.S. Food and Drug Administration (FDA), the European Medicines Agency (EMA), and national health authorities in various countries require 3D-printed dental devices to meet rigorous criteria before they can be used in clinical settings [56]. However, because 3D printing is an emerging technology with rapidly evolving applications, regulatory pathways often lag behind technological advances, creating a complex landscape for manufacturers and dental practices [4].

One of the primary regulatory challenges is the classification of 3D-printed dental devices due to their unique manufacturing process and patient-specific designs. According to Ricles et al. [84], the FDA must evaluate these devices within specific material and safety standards, especially since dental devices often use biocompatible polymers that require rigorous testing for intraoral use and durability [84].

The regulatory pathway, typically the 510(k) premarket notification, demands that these devices be proven as safe and effective as existing legally marketed counterparts; however, finding comparable predicates for highly customized, patient-specific designs can be complex. Additional challenges include maintaining quality and consistency across varying anatomical designs to ensure these custom devices function as intended [86]. Furthermore, robust post-market surveillance, including adverse event tracking, is necessary to monitor any issues related to design or material failures over time. These challenges highlight the FDA’s need for adapted regulatory frameworks to address the intricacies of additive manufacturing in the dental field [87]. Also, The European Union has rigorous standards for 3D-printed dental devices under the Medical Device Regulation (MDR), which necessitate clinical evidence demonstrating safety and performance [88].

Furthermore, there are several technical limitations, 3D printing technology faces challenges related to printer resolution, print times, and post-processing requirements [61]. While many 3D printing technologies, such as SLA and DLP, offer high resolution suitable for dental applications, limitations still exist in achieving the fine detail and smoothness required for certain intricate structures [89]. Additionally, the time required for printing and post-processing can be substantial, particularly for larger or more complex models [56].

Adoption barriers also play a significant role in determining the extent to which 3D printing can be integrated into dental practices, particularly for small or low-resource clinics [56]. Smaller practices may struggle to justify or afford the upfront expenses associated with 3D printing, leading to disparities in the quality of dental care available to different patient populations [4].

## Future Perspectives and Emerging Trends

As 3D printing technology continues to evolve, several emerging trends and advancements are expected to further enhance its application in dentistry [2]. Innovations in materials, integration with artificial intelligence (AI), patient-centered personalization, and sustainability efforts are all anticipated to shape the future of 3D-printed dental devices, providing improved outcomes and greater accessibility [81].

Material advancements represent one of the most promising areas of progress in 3D printing for dentistry [90]. The development of new biocompatible materials with enhanced durability, aesthetics, and functional performance has the potential to address some of the current limitations in dental applications [91]. Future materials may include hybrid resins and composite polymers that combine strength with a natural appearance, suitable for high-stress applications such as crowns and bridges [67]. Moreover, bioactive materials that can promote tissue integration or reduce bacterial adherence are being explored to support long-term oral health and device longevity [1]. Innovations in metal powders for SLS could also yield stronger, lighter, and more corrosion-resistant options for dental implants, enabling greater functional durability and patient satisfaction [92].

The integration of AI with 3D printing is another emerging trend that holds significant potential for optimizing the design and production of dental devices [81]. AI can be used to analyze patient-specific data, such as digital scans and radiographic images, to create highly accurate and optimized models for prosthetics and orthodontic appliances [67]. By automating the design process, AI can reduce human error, enhance precision, and enable faster and more cost-effective production of 3D-printed devices [90]. Furthermore, machine learning algorithms can analyze patterns across large datasets of patient outcomes, enabling continuous improvement in the design of dental devices [81].

Personalized medicine is increasingly becoming a focal point in healthcare, and 3D printing is at the forefront of advancing patient-centered, personalized dental care [6]. Through digital imaging and customization capabilities, 3D printing allows dental practitioners to design devices tailored to the individual anatomy and functional requirements of each patient [2]. This degree of customization not only improves the comfort and fit of prosthetics and orthodontic appliances but also aligns with the broader shift toward personalized treatment strategies in dentistry [91]. 3D printing can also facilitate the production of devices that address specific patient needs, such as pediatric prosthetics, age-related dental issues, or devices for individuals with complex medical histories [67].

Finally, the environmental impact of 3D printing in dentistry is an area of growing importance, especially as sustainability becomes a priority across industries [7]. Many 3D printers are now compatible with eco-friendly, recyclable, or biodegradable materials, further supporting environmentally conscious practices [70]. Future developments in material recycling within 3D printing processes could enable dental practices to minimize their environmental footprint, promoting waste reduction and sustainable resource use [71].

## Conclusion

3D printing has emerged as a transformative technology in the field of dentistry, offering remarkable benefits in the customization and efficiency of dental prosthetics and orthodontic appliances. Through its ability to create highly individualized devices tailored to patient-specific anatomy, 3D printing enhances the precision, fit, and comfort of dental prosthetics such as crowns, bridges, and dentures, as well as orthodontic devices like aligners and retainers. By reducing the reliance on traditional manufacturing methods, which are often labor-intensive and time-consuming, 3D printing enables faster production and lower operational costs for dental practices. This shift towards digital and additive manufacturing provides not only improved patient outcomes but also a more streamlined workflow for clinicians, ultimately fostering greater accessibility and responsiveness in dental care.

The implications of 3D printing in dentistry extend beyond immediate clinical applications. As technology and materials continue to advance, dental practices are likely to see increased integration of digital workflows and personalized care, making sophisticated treatments more available to a wider range of patients. Future research will play a critical role in overcoming existing limitations, such as material constraints and regulatory challenges, to further expand the capabilities of 3D printing in the dental sector. Continued innovation in biocompatible materials, AI-driven design optimization, and environmentally sustainable practices will shape the next generation of dental solutions, offering promising avenues for both clinical enhancement and cost-effectiveness. As such, 3D printing stands poised to redefine modern dentistry, providing a foundation for more effective, patient-centered, and accessible dental care in the years to come.

## Conflict of Interest

None declared.
